# Targeted Six-Week Intensive Physiotherapy for a Case of Tuberculous Meningitis With a Syndrome of Inappropriate Antidiuretic Hormone Secretion

**DOI:** 10.7759/cureus.55214

**Published:** 2024-02-29

**Authors:** Arjavi A Pakhan, Raghuveer Raghumahanti

**Affiliations:** 1 Neurophysiotherapy, Ravi Nair Physiotherapy College, Datta Meghe Institute of Higher Education and Research, Wardha, IND

**Keywords:** miliary tuberculosis, siadh, physical therapy, multiple tuberculoma, tubercular meningitis

## Abstract

Tuberculous meningitis (TBM) is a severe form of extrapulmonary tuberculosis (TB) characterized by the invasion of *Mycobacterium tuberculosis* into the meninges surrounding the brain and spinal cord. It triggers an intense inflammatory response, leading to neurological complications if not promptly and adequately managed. TBM often precipitates muscle weakness, neurological deficits, respiratory challenges, swallowing difficulties, joint contractures, and pain. Physiotherapy intervention is essential in treating these problems by personalized treatment strategies and treatment plans to enhance muscle strength, motor control, coordination, and overall mobility. This case report aims to highlight the significant role of physiotherapy in improving the quality of life (QOL) and functional abilities of patients with TBM. The current case report reviews the case of a 73-year-old male who presented with complaints of generalized weakness and difficulty in swallowing. The patient had a history of fever for the last six months. Magnetic resonance imaging (MRI) and high-resolution computed tomography (HRCT) diagnosed the case as TBM with miliary TB. Six weeks of targeted intensive rehabilitation program was designed according to the patient's impairments initiated from the intensive care unit (ICU) phase. The main goals of physiotherapy were to start early bed mobility, maintain joint integrity, improve postural strength and swallowing, and make the patient independent in transfer and activities of daily living (ADLs). After a six-week intensive physiotherapy (TIP-6) program, the patient exhibited significant improvements in muscle strength and independence in ADLs. This case highlights the critical role of physiotherapy in enhancing the QOL and functional abilities of patients with severe TB-related conditions.

## Introduction

Tuberculous meningitis (TBM), miliary tuberculosis (TB), and multiple tuberculomas represent distinct yet interconnected clinical entities, each stemming from the insidious *Mycobacterium tuberculosis* infection. These TB manifestations reflect this pathogen's remarkable ability to disseminate through the bloodstream and lymphatic system, causing diverse clinical presentations with intricate diagnostic and therapeutic challenges [1]. TBM is one of the most common and serious forms of TB in India. India has the highest burden of TB in the world, accounting for over 25% of global cases. Syndrome of inappropriate antidiuretic hormone secretion (SIADH) is a known complication of TBM, occurring in 5-30% of cases. *Mycobacterium tuberculosis* infiltrates the meninges around the brain and spinal cord, resulting in a severe type of extrapulmonary TB known as TBM. This invasion triggers an intense inflammatory response, leading to neurological complications if not promptly and adequately managed [2]. TBM is notorious for its subtle initial symptoms, which often include fever, headache, and altered mental status, progressing to more severe manifestations such as cranial nerve deficits and focal neurological signs [3]. Early diagnosis and initiation of anti-tubercular therapy are pivotal for favorable outcomes, as delays can result in significant morbidity and mortality [4].

Hematogenous spread tuberculous bacilli, which spread widely throughout the body and produce multiple tiny granulomas in different organs, are the hallmarks of miliary TB. Numerous organ systems can be impacted by these minute granulomas, often known as "miliary" lesions, which cause the condition's varied clinical presentations [5]. Individuals may have systemic signs that resemble other infectious or malignant processes, such as fever, weight loss, and sweats at night [6]. Because of its complex clinical picture, miliary TB is often difficult to diagnose. On the other hand, multiple tuberculomas, which typically affect the brain and lungs, are localized accumulations of *Mycobacterium tuberculosis* within tissues or organs [7]. These tuberculomas may cause localized neurological impairments or pulmonary symptoms, depending on where they are anatomically located [8]. Sometimes, TBM or miliary TB coexists with multiple tuberculomas, complicating the clinical presentation and diagnostic assessment [9,10].

The ensuing cascade of events disrupts the blood-brain barrier, forms caseating granulomas, and releases pro-inflammatory cytokines, ultimately leading to neurological damage [8]. TBM is diagnosed through a combination of clinical assessment and laboratory tests. Lumbar puncture is used to analyze the cerebrospinal fluid (CSF) for biochemical signs of TB infection such as low glucose, elevated proteins, and the presence of TB bacteria. Neuroimaging like computed tomography (CT) or magnetic resonance imaging (MRI) scans can reveal basilar meningeal inflammation indicative of TB infection. Microbiological tests like GeneXpert on the CSF can provide rapid evidence of TB bacteria. Evaluating for accompanying pulmonary TB through chest imaging and sputum evaluation is important for diagnosing and treating TBM. Assessing for exposure history or immunological evidence of latent TB is critical for determining if the meningeal TB represents primary infection or reactivation of old infection. Clinical findings like fevers, mental status changes, and hydrocephalus also aid diagnosis [11]. Physiotherapy is crucial in the multidisciplinary approach to treating patients with TBM [12]. TBM often inflicts severe neurological dysfunction in patients, causing muscle weakness, sensory disturbances, and cognitive impairments [13]. In the comprehensive management of such complications, physiotherapy plays a crucial component in rehabilitation, focusing on physical aspects to restore functional independence and overall quality of life (QOL) [14]. Physiotherapy uses various methods to treat patients with motor deficits, including passive and active range of motion (ROM) exercises [15]. This is important because it prevents complications like joint stiffness and muscle contractures, which are frequently seen in individuals who have been immobilized. Physiotherapy aids in the restoration of mobility and functional independence by progressively increasing the ROM [5].

Moreover, physiotherapists focus on enhancing gait and balance, which are problems that individuals with neurological dysfunction often face. TBM survivors often experience challenges in walking and maintaining proper posture, making them susceptible to falls and related injuries [16]. Through specialized training and exercises, physiotherapy is pivotal in helping patients achieve optimal balance and gait patterns, ultimately enhancing their overall mobility. Sensory deficits represent another neurological issue encountered by TBM patients. These deficits can significantly diminish patients' QOL. Physiotherapists employ sensory re-education techniques to assist patients in regaining sensory perception, such as touch and proprioception. These methods are precious in reinstating normal function and reducing discomfort associated with sensory deficits [17]. The main aim is to highlight the significant role of physiotherapy in improving QOL and functional abilities in a 73-year-old male diagnosed with TBM. A six-week targeted intensive rehabilitation program was initiated based on the patient's impairments.

## Case presentation

Patient information

A 73-year-old male with right-hand dominance was admitted to the neurology intensive care unit (ICU) with complaints of generalized weakness and difficulty in swallowing. The patient had a history of recurrent fever occurring every month for the past six months and visited a private clinic where paracetamol was prescribed to him. In September, the patient's condition worsened as he started experiencing severe headaches, recurrent fever, chills, generalized weakness, and body pain. Subsequently, the patient was admitted for further evaluation.

Clinical findings

**Figure 1:**
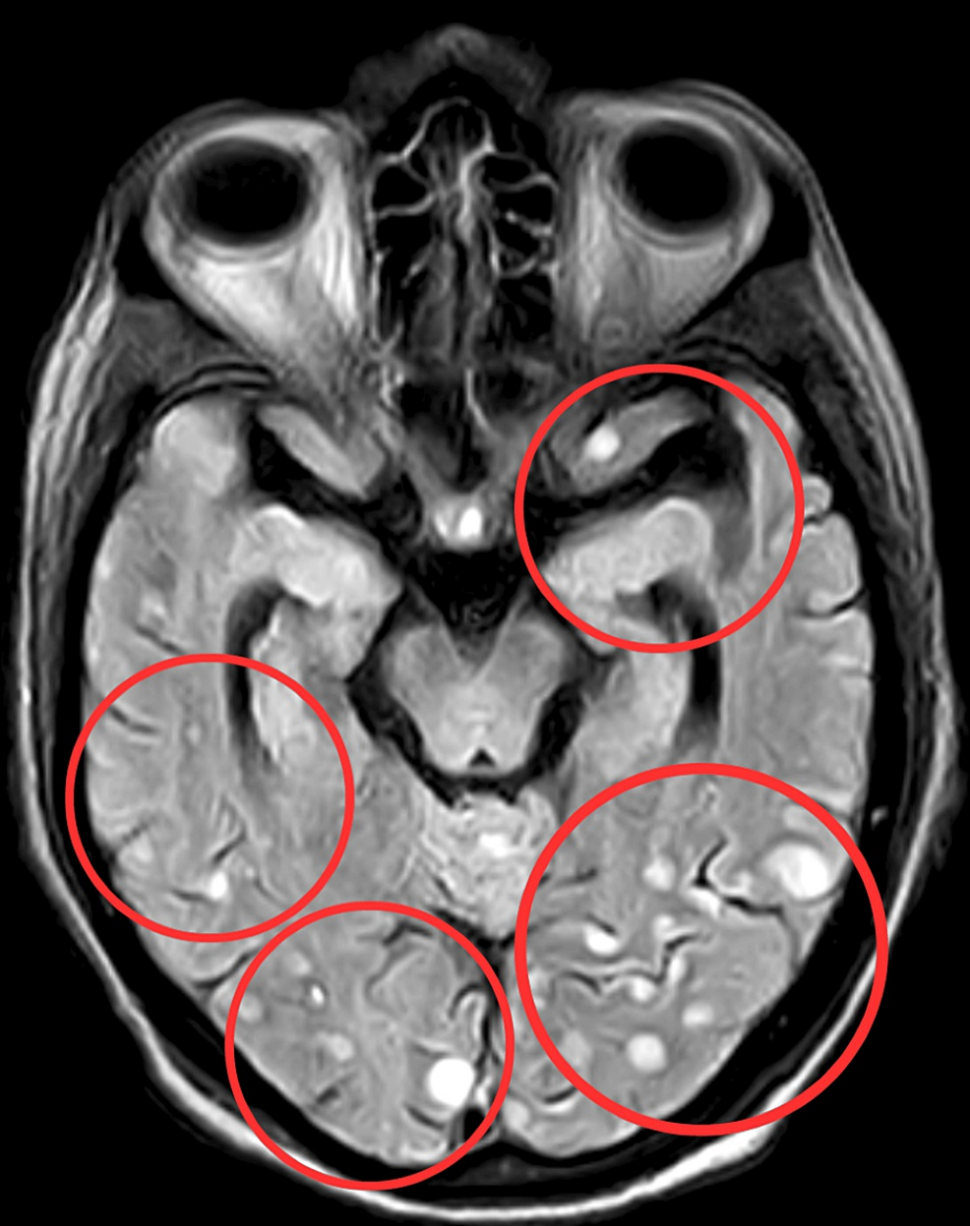
MRI of the brain The red circles show variable-sized round to oval lesions noted in the bilateral cerebral hemisphere and bilateral cerebellar hemisphere MRI: magnetic resonance imaging

The first examination was done following the patient's and family's consent. The patient was conscious, cooperative, and well-oriented to time, place, and person. The patient's attitude of the limb was in supine lying, and the upper limb exhibited slight shoulder abduction with elbow flexion and a neutral wrist position while in a supine position. The lower limb showed external hip joint rotation, extended knee, and ankle plantarflexion. The Glasgow Coma Scale (GCS) was E4V5M6 on higher mental function evaluation. The patient was connected to external medical appliances, including a Foley catheter and a Ryles tube. On the sensory examination, the sensory functions in the upper extremities were intact, while superficial reflexes, including fine touch and pin-prick, were absent in the lower extremities. On motor examination, the muscle tone of the upper and lower extremities was normal. According to the Oxford grading system, manual muscle testing (MMT) showed 3/5 in the upper limb, which is full ROM against gravity without resistance; in the lower limb, it was 2/5, which is full ROM in gravity eliminated, shown in Table 1. Kernig's sign was positive. High-resolution CT (HRCT) of the thorax reveals diffusely scattered miliary nodules in bilateral lung parenchyma with a patch area of consolidation in the left lower lobe and mediastinal lymphadenopathy-miliary TB. MRI of the brain reveals evidence of variable-sized round to oval lesions noted in the bilateral cerebral hemisphere and bilateral cerebellar hemisphere. The lesions appear hypointense on TI WI and hyperintense on T2 WI/FLAIR, showing blooming on SWI and no restriction on DWI suggestive of (s/o) partial calcification. There is the prominence of sulco-gyral space, ventricular system, Sylvian fissure, and cerebellar folia s/o age-related atrophic changes, as shown in Figure 1. 

**Table 1:** MMT MMT: manual muscle testing

MMT	Pre-treatment			Post-treatment		
Muscles	Right		Left	Right		Left
Shoulder						
Flexors	3/5		3/5	4/5		4/5
Extensors	3/5		3/5	4/5		4/5
Abductors	3/5		3/5	4/5		4/5
Adductors	3/5		3/5	4/5		4/5
Elbow						
Flexors	3/5		3/5	4/5		4/5
Extensors	3/5		3/5	4/5		4/5
Wrist						
Flexors	3/5		3/5	4/5		4/5
Extensors	3/5		3/5	4/5		4/5
Hip						
Flexors	2/5		2/5	3/5		3/5
Extensors	2/5		2/5	3/5		3/5
Abductors	2/5		2/5	3/5		3/5
Knee						
Extensors	2/5		2/5	3/5		3/5
Flexors	2/5		2/5	3/5		3/5
Ankle						
Dorsiflexors	2/5	2/5		3/5	3/5	
Plantarflexors	2/5	2/5		3/5	3/5	

Therapeutic intervention

**Figure 2:**
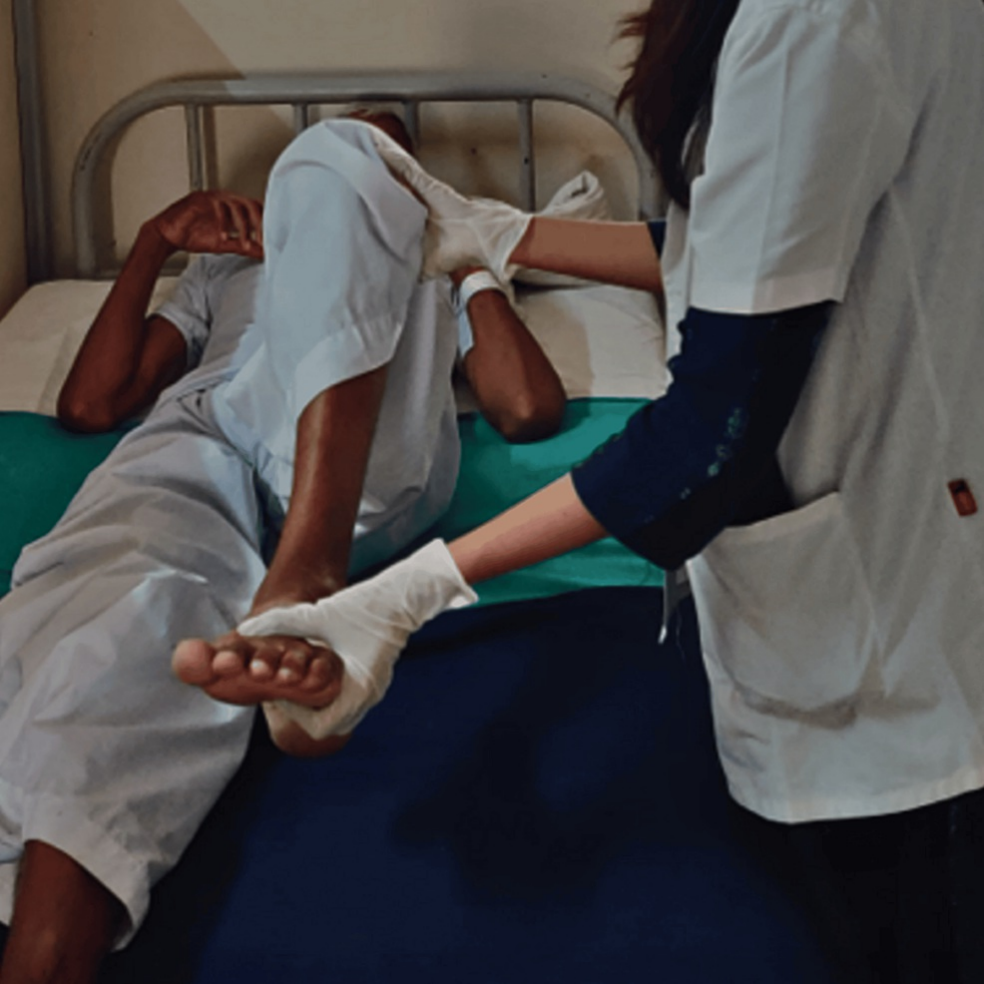
Passive ROM exercise ROM: range of motion

**Figure 3:**
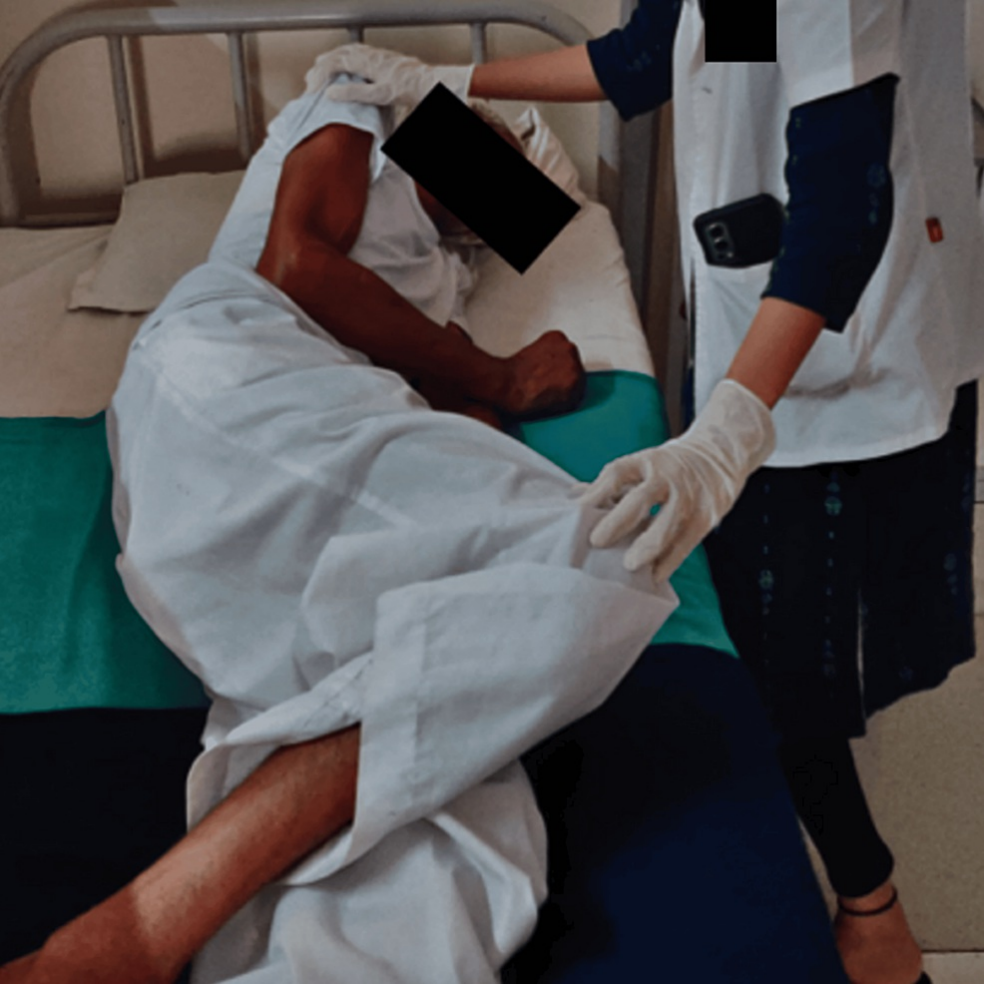
Rolling facilitation

A targeted six-week intensive physiotherapy (TIP-6) intervention was started as soon as the patient was in ICU. Table 2 shows the treatment protocol given to the patient for six weeks. Figure 2 and Figure 3 show the patient being rehabilitated.

**Table 2:** Targeted six-week intensive physiotherapy DVT: deep vein thrombosis, QOL: quality of life, ROM: range of motion, PNF: proprioceptive neuromuscular facilitation, ADLs: activities of daily living

Problems faced by the patient	Goals	Intervention			Rationale
		0-2 weeks	2-4 weeks	4-6 weeks	
Respiratory complications	To prevent respiratory complications	Initiate deep breathing exercises, targeting the diaphragmatic and thoracic regions, involving inhalation and exhalation for three sets of 10 breaths. In week 2, further introduce longer breath-hold durations to enhance respiratory endurance	Additional deep breathing techniques (diaphragmatic and pursed lip breathing) to further enhance respiratory capacity	Incentive spirometry, to further enhance respiratory capacity	Increase the oxygen level. Help restore the diaphragmatic function of muscle
Other secondary complications	To prevent bed sores, contracture, and circulatory problems	Repositioning every two hours to prevent pressure ulcers and DVT. Employed supportive pillows and sandbags. In week 2, continue these practices while educating the patient on self-repositioning techniques to enhance engagement and maintain optimal limb elevation	Continue emphasizing proper positioning every two hours to prevent pressure ulcers and DVT. Reinforce the importance of limb elevation and educate the patient on self-repositioning techniques	Deep breathing exercises and effective coughing techniques. Regular practice of these techniques is emphasized to prevent chest complications	To prevent DVT, contracture, and circulatory complications
Muscle weakness	To improve muscle strength	Initiated isometric exercises for key muscle groups (biceps, triceps, deltoid, quadriceps) at a light to moderate intensity. Perform 1-2 sets of 10-15 repetitions. In week 2, maintain the current exercises with the same resistance level or consider progressing to bodyweight squats or step-ups for the lower limb muscles	Maintain current strength training exercises with a focus on the lower limb muscles. Perform two sets of 10-15 repetitions. In week 3, consider introducing bodyweight squats or step-ups for the lower limbs. Exercises are conducted at a light to moderate intensity	Progress the lower limb strength training exercises by introducing resistance bands or light dumbbells. Focus on exercises that target the quadriceps, hamstrings, glutes, and calf muscles. Perform 2-3 sets of 10-15 repetitions at a light to moderate intensity	Enhance the QOL. Enhance mobility pain management
Difficulty in movement	To maintain joint integrity	Passive and active assisted movements for bilateral upper and lower limbs, consisting of 10 repetitions for each movement to enhance joint ROM. In week 2, maintain these exercises while gradually increasing the number of repetitions to challenge the patient's mobility	Continue mobility with joint exercises while considering more advanced procedures, such as pendulum swings and joint mobilization techniques	Continue with joint mobility exercises and introduce advanced techniques like contract-relax stretching and PNF to further enhance the ROM	Improve muscle strength and prevent joint stiffness
Difficulty in transfer	To initiate bed mobility and to make patient transfer	Mobility training-bed rolling, bedside sitting, and wheelchair mobility exercises aimed at promoting functional independence	Continue with mobility training and make it more challenging to help the patient become more independent and safer in everyday tasks	Maintain the current mobility exercises and introduce more challenging activities that simulate daily tasks, including step-ups and controlled walking on even surfaces	Maintain their independence in ADLs and improve mobility
Poor balance and coordination	To balance independently	Balance training with a 30-minute elevation of the bed head to challenge balance and stability. In week 2, maintain this regimen while incorporating more advanced balance exercises, such as single-leg balance and proprioception activities	Maintain the 30-minute bed head elevation and incorporate more challenging balance exercises, such as tandem stance and proprioception activities, to further enhance stability and coordination	Continue with balance exercises and advance to dynamic balance activities, like walking on uneven surfaces or using balance boards, to further enhance stability and coordination	Improve stability, to reduce the risk of fall
Sitting difficulty	To sit independently	Assess the patient's sitting difficulty and initiate bedside sitting with maximum support for five minutes. In week 2, continue with bedside sitting while progressively reducing support, aiming for increased independence	The duration to build the patient's sitting endurance. In week 3, work on achieving independent sitting without any support	Focus on achieving independent sitting without support. Gradually increase the duration of sitting exercises to enhance endurance and overall functional independence	Sitting independently without any support
Difficulty in swallowing	To improve swallowing	Changing food textures, optimizing posture during meals, and using adaptive tools	Based on patient feedback and needs, optimize food textures, posture during meals, and adaptive tools to enhance the swallowing process	Incorporate more complex food textures and optimize mealtime posture	Enhancement of nutritional status. To prevent aspiration. To prevent choking

Outcome measures 

The TIP-6 was carried out for three weeks following the progression outcome measures, which showed improvement after the physiotherapy intervention. Outcome measures are mentioned in Table 3.

**Table 3:** Outcome measures ICU: intensive care unit

Outcome measures	Pre-treatment	Post-treatment
Functional Outcome Swallowing Score	3/4	1/4
Barthel Index of Activities of Daily Living Scale	0/0	40/100
Expanded ICU Mobility Scale and Intensity Classification	0/6	4/6
Quality of Life Scale	57/112	69/112

## Discussion

In TBM, the inflammatory response often leads to muscle weakness and neurological deficits. Physiotherapy interventions play a pivotal role in improving muscle strength and mobility. By engaging in targeted exercises such as isometric muscle training and bodyweight squats, the patient regains muscular strength and re-establishes neural pathways. This neurological reconnection, facilitated by physiotherapy, aids in reversing muscle atrophy and promoting coordinated muscle function, ultimately countering the muscle weakness seen in TBM. Physiotherapy effectively addresses respiratory problems that may occur due to reduced lung function and weaker respiratory muscles in patients with TBM. Exercises that involve deep breathing, incentive spirometry, and other breathing methods aid in increasing lung capacity and regaining the function of the diaphragmatic muscle. Physiotherapy enhances the muscles that control breathing, increases endurance, and improves oxygen exchange. As a result, this intervention improves the patient's respiratory health in general, helping to improve oxygenation and avoiding problems such as pneumonia. Physiotherapy can help with balance and coordination problems, mobility challenges, and transfer difficulties. The exercises promote neural plasticity, allowing the patient to regain functional independence and improve overall mobility. Additionally, balance training fosters neural adaptations, enhancing stability and coordination, thus reducing the risk of falls. Physiotherapy's modifications to food textures and mealtime posture, combined with adaptive tools, help the patient improve their swallowing ability. By optimizing these aspects, physiotherapy ensures that the patient receives proper nutrition and reduces the risk of aspiration. Physiologically, these interventions enhance the coordination of swallowing muscles and reduce the chances of food or liquids entering the airway, ultimately preventing choking and improving the patient's overall nutritional status.

As demonstrated by critical measurements, targeted intensive physiotherapy intervention made a significant difference in the patient's health. Initially, at 0/100, the Barthel Index increased to 40/100 after treatment, showing a substantial improvement in the patient's ability to do daily tasks without assistance. Furthermore, the Functional Outcome Swallowing Score (FOSS) improved from 3/4 to 1/4, indicating substantial progress in the patient's ability to swallow safely without the risk of aspiration. These results highlight the comprehensive impact of physiotherapy on the patient's overall well-being, encompassing gains in muscle strength, mobility, respiratory health, and ADLs. The results of this case report highlight the importance of incorporating rehabilitation programs into the management of TBM patients. Early initiation of rehabilitation, even while patients are in the ICU, can lead to improved outcomes and reduced disability.

The results align with similar studies on rehabilitating TBM patients. Suisan and Thohari presented two severe TBM cases who received a multimodal sensory stimulation program incorporating positioning, ROM exercises, and tactile and auditory stimuli during their ICU stay. After two weeks, both patients showed remarkable progress with GCS scores improving from E1V1M1 to E4V5M6, allowing transfer from ICU to high-dependency unit. The intensive early rehabilitation likely contributed to their neurological and functional improvements [18].

In a study by Wang et al., they discussed a case of an immunocompetent patient who had a brain abscess caused by TB. This patient developed problems like Gerstmann's syndrome and right-sided apraxia. However, following TB treatment and rehabilitation, the patient made a full recovery, resumed their regular activities, and even went back to work. This case highlights the importance of intensive rehabilitation, precise surgery (stereotactic surgery), and effective TB treatment when dealing with such conditions. However, after a meticulously thought-out six-week rehabilitation program, notable progress was observed, including the patient's ability to sit independently without assistance [7].

## Conclusions

In this case, the patient had TBM with multiple tuberculoma with miliary TB with SIADH, and physical therapy was started when the patient was in the ICU. TIP-6 interventions improved the patient's abilities and independence while preventing respiratory and secondary complications. Total muscle strength had all improved significantly, as shown in the above scales. Physiotherapy helps the patient to enhance his ADLs and QOL.
